# Estrogen therapy offsets thermal impairment of vitellogenesis, but not zonagenesis, in maiden spawning female Atlantic salmon (*Salmo salar*)

**DOI:** 10.7717/peerj.3897

**Published:** 2017-11-02

**Authors:** Kelli Anderson, Ned Pankhurst, Harry King, Abigail Elizur

**Affiliations:** 1 Genecology Research Centre, Faculty of Science, Health, Education and Engineering, University of the Sunshine Coast, Sippy Downs, Queensland, Australia; 2 Australian Seafood Cooperative Research Centre, Bedford Park, South Australia, Australia; 3 Australian Rivers Institute, Griffith University, Gold Coast, Queensland, Australia; 4 Commonwealth Scientific and Industrial Research Organisation, Hobart, Tasmania, Australia

**Keywords:** Gene expression, Temperature, Salmon, Ovarian steroidogenesis, Climate change, Aquaculture, Hormonal therapy

## Abstract

In female Atlantic salmon (*Salmo salar*), exposure to warm summer temperatures causes a reduction in plasma 17β-estradiol (E2), which impairs downstream vitellogenesis and zonagenesis, and reduces egg fertility and embryo survival. The aim of the present study was to determine whether E2-treatment could offset thermal impairment of endocrine function and maintain egg quality in maiden (first-time-spawning) * S. salar* reared at 22 °C. Treatment with E2 at 22 °C stimulated vitellogenin (*vtg*) gene expression and subsequent protein synthesis which promoted oocyte growth and increased egg size relative to untreated fish at 14 and 22 °C. However, E2-treatment at 22 °C was not associated with an increase in egg fertility and embryo survival relative to untreated fish at 22 °C, despite the positive effects of E2-treatment on vitellogenesis and oocyte growth. As there was no evidence to suggest that the estrogen receptor alpha expression was suppressed by high temperature, this could be due to the lack of stimulation on zonagenesis by E2-treatment observed at high temperature during oocyte development. Our results demonstrate that treatment with E2 is not able to maintain zonagenesis or egg quality in maiden * S. salar* at high temperature, even when * vtg* gene expression, protein synthesis and subsequent oocyte growth is promoted. This implies that the mechanisms regulating zonagenesis, but not vitellogenesis are impaired at elevated temperature in female * S. salar* broodstock, and highlights the remarkable complexity of thermally induced endocrine disruption in fish.

## Introduction

Tasmanian Atlantic salmon (*Salmo salar*) are currently reared towards their upper-limit of thermal tolerance for successful reproduction (King & Pankhurst, 2003), and can sometimes experience temperatures up to and exceeding 20 °C during summer months (Battaglene et al., 2008). The negative effects of thermal exposure on reproductive processes are mediated through the brain-pituitary-gonad axis, and endocrine function can be impaired at multiple levels. For example, thermal impairment occurs at the level of the ovary in female pejerrey (*Odontesthes bonariensis*), as expression of follicle stimulating hormone receptor (*fshr*) and plasma 17β-estradiol (E2) levels are suppressed at high temperatures (Soria, Strüssmann & Miranda, 2008). While a recent study by Anderson et al. (2017) found no evidence of *fshr* inhibition at 22 vs 14 °C in adult *S. salar* during vitellogenesis, the suppressive effects of high temperature on ovarian steroidogenesis are well documented. For example, suppression of plasma E2 and testosterone (T), and expression of P450 aromatase A (*cyp19a1a*), cholesterol side chain cleavage protein (*cyp11a1*)*,* steroidogenic acute regulatory protein (*star*)*,* and 3β-hydroxysteroid dehydrogenase (*3β-hsd*) are a typical feature of thermally exposed female *S. salar*, and as a consequence downstream production of maturation-inducing steroid (17,20β-dihydroxy-4-pregnen-3-one, MIS), vitellogenin (Vtg), and hepatic expression of *vtg*, zonapellucida c (*zpc*) and b (*zpb*) are impaired (Anderson et al., 2017; Anderson et al., 2012a; King & Pankhurst, 2004; Pankhurst et al., 2011). For salmonids, endocrine impairment associated with exposure to elevated temperatures consistently leads to a reduction in egg fertility and embryo survival (King et al., 2003; Pankhurst et al., 1996; Taranger & Hansen, 1993), and the effects are typically more pronounced in maiden (first-time) spawning *S. salar* (Pankhurst et al., 2011). This indicates that the salmon industry must adapt to ensure the production of high quality smolts, as periods of high temperature become more frequent due to climate change (Pankhurst & King, 2010).

During production, the use of repeat (second-time-spawning) fish over maidens is not a desirable option due to the considerable cost and risk associated with intensively maintaining such large fish for an additional year. An alternate management option may be to optimise egg production in maidens by administering one or more exogenous hormones that will, in theory, mitigate the negative effects of thermal exposure on endocrine function and therefore maintain egg quality (Pankhurst & King, 2010). Gonadotropin releasing hormone analogue (Gnrha) injection in combination with a temperature reduction prior to ovulation was able to fully restore egg fertility in *S. salar* reared at 16 °C during late vitellogenesis (King & Pankhurst, 2004). However, there are multiple endocrine ‘blockages’ that occur at the level of ovarian steroidogenesis during vitellogenesis as mentioned, and for this reason administration of Gnrha during peak-vitellogenesis may not be effective. Alternatively, E2 (or 17α-ethynylestradiol, EE2) treatment has been shown to induce *de novo* synthesis of Vtg at elevated temperatures in juvenile salmonids (Anderson et al., 2012b; Körner et al., 2008; Westerlund et al., 2001). Thus, administration of E2 may surpass *cyp19a1a* and other impairments in the ovary, and directly stimulate the downstream synthesis of Vtg at high temperature.

Given the industry’s preference to use maiden fish for egg production, the aim of the present study was to determine whether E2-therapy in maiden broodstock could overcome the effects of reduced endogenous E2, and maintain egg quality at 22 °C. Accordingly, the effects of E2-therapy (exposure to E2 in combination with the process of implantation) on oocyte development and endocrine function were determined, including plasma Fsh, luteinizing hormone (Lh) and Vtg levels, and hepatic gene expression levels of *vtg*, *zpb*, *zpc* and estrogen receptor alpha (*erα*) during vitellogenesis. Thereafter, the effects of hormonal and thermal manipulation on the timing of ovulation and subsequent egg fertility and embryo survival were assessed.

## Materials & Methods

### Fish husbandry and maintenance

**Figure 1:**
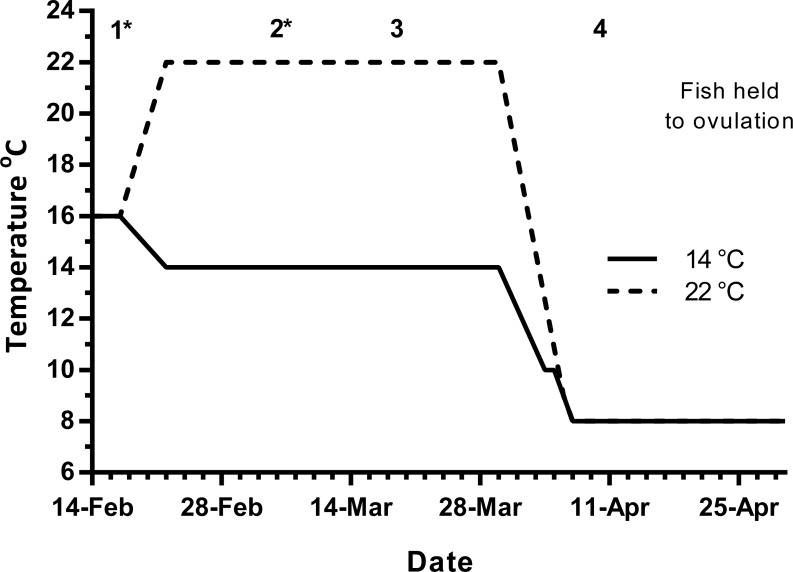
Thermal treatment regimes, hormone pellet implant dates (indicated by asterisk) and sampling times (1–4) for maiden *S. salar* held at 14 or 22 °C in late summer and autumn.

Maiden (2 + year old) adult females were held in 200 m^3^ circular tanks at ambient photoperiod and temperature, at the Salmon Enterprises of Tasmania Wayatinah hatchery until February 2010. On the 17th of February, fish were transferred to temperature-controlled 4 m^3^ Rathbun tanks (14 fish per tank) under simulated ambient photoperiod according to the following 3 treatment groups: (1) 14 °C untreated, (2) 22 °C untreated and (3) 22 °C + E2 implant. Fourteen and 22 °C represent cool and warm Tasmanian summers respectively. Fish were not fed from the time of transfer to the temperature controlled systems in January consistent with hatchery practice for management of this stock of fish. All fish were maintained at the nominated temperature until late March, when they were exposed to a gradual temperature ramp down to 8 °C to induce final oocyte maturation and ovulation approximately 1 week before the last sample point as described by King & Pankhurst (2000) (Fig. 1).

Early 2010 was particularly warm, with the average water temperature in late January, and early February reaching 20.5 and 21.0 °C respectively. During this period of high air temperatures and very low rainfall, a bush fire spread through Wayatinah, and caused a delay to the experiment start date. As such, ambient water temperatures during the experimental period were approximately 1.5 (January) and 2.0 °C (February) warmer than the conditions experienced by stock from our previous work (Anderson et al., 2017; Anderson et al., 2012a; Pankhurst et al., 2011).

### Sampling protocol

Fish maintained at ambient temperature were sampled (*n* = 7) on February 17th (sample 1) to establish a vitellogenic base line for the rest of the experiment (Fig. 1). Then on March 5th (sample 2), March 19th (sample 3) and on April 9th (sample 4), seven fish from each group were sampled leaving seven fish from each treatment to progress through to ovulation and stripping in May–June. Fish in group 3 were given E2 pellets at samples 1 and 2. For implantation, fish were anaesthetised in Aqui-S™ and a small ventral incision was made with a scalpel, the pellet was then inserted using forceps and the wound sealed with medical adhesive (Stoma Powder).

For sampling, fish were netted from the holding tanks, terminally anaesthetised in Aqui-S ™ (Crop & Food, Lower Hutt, New Zealand), weighed, measured and then blood sampled by caudal puncture using pre-heparinised syringes fitted with 22 G needles. Blood was centrifuged at 12,000 g, and plasma stored at −20 °C. Ovaries were excised, weighed and portions allocated to 50 ml pots containing teleost saline for fecundity estimation and follicle measurement. A segment of ovarian tissue was also fixed in 10% neutral buffered formalin for later histological examination. Segments of liver were transferred to 1–2 ml of RNA Later™ (Qiagen, Hilden, Germany), kept at 4 °C overnight, and then stored at −80 °C.

Gonadosomatic indices (GSI) were calculated as (gonad weight/total body weight) × 100, and condition factor (CF) as (body weight/length^3^) × 100. Fecundity was determined by dispersing all ovarian follicles from an ovarian segment of ∼5 g in teleost saline then counting all vitellogenic (opaque) follicles present in the sample. Total fecundity was determined by correction for total ovarian weight and expressed as relative fecundity kg^−1^ body weight. At ovulation, ova were stripped, fertilised, and incubated under the same conditions as described in King et al. (2003) for measurement of egg size, fertility and survival to the eyed stage at 250 degree-days of incubation. All animal experiments were conducted in accordance with Australian law under ethical approval issued by the Griffith University and University of the Sunshine Coast Animal Ethics Committees (ENV/01/10/AEC and AN/A/10/50 respectively).

### E2 implants

Pellets containing E2 were manufactured by combining 35 mg of crystalline E2 with 500 μl of unpolymerised silastic elastomer (Dow Corning, Midland, MI, USA), adding accelerant, and then centrifugation in 1.5 ml Eppendorf tubes to provide solid cured pellets. Each fish (approximate weight 3.5 kg) received one pellet at each treatment to give a notional dose of 10 mg kg^−1^.

### Plasma hormone and vitellogenin measurement

Plasma Fsh (all sampling points) and Lh (late March and April only) measurements were performed using an RIA developed for coho salmon (*Oncorhynchus kisutch*) by Swanson et al. (1989) with some modifications. Briefly, the assay utilised rabbit antisera specific to the *O. kisutch* Fsh or Lh beta subunit (lots #8621 and #38.5.92 respectively), and highly purified *O. kisutch* Fsh and Lh as the standards (Swanson et al., 1991). In these assays, phosphate buffered saline (pH 7.4) was used instead of barbital, and 500 μl polyethylene glycol (4%) was included on day 4. The cross reactivity of Fsh in the Lh assay, and Lh in the Fsh assay was approximately 4.4 and 6%, respectively (Swanson et al., 1989). ANCOVA was performed as part of a previous study to determine whether parallelism was present between *S. salar* plasma and the respective purified *O. kisutch* standard that was serially diluted in PBS-BSA (Anderson et al., 2012a). The lower detection limits (LOD) of the Fsh and Lh assays were approximately 0.6 and 0.5 ng ml^−1^ respectively.

Plasma levels of E2, T and cortisol (F) were determined by radioimmunoassay. E2 was extracted from 100 µl of plasma using 1 ml ethyl acetate, the reagents and procedure for E2 and T described in Pankhurst & Carragher (1992), and for F as in Pankhurst et al. (2008). Extraction efficiency was 78, 79 and 74% for E2, T and F respectively as determined by recovery of ^3^H-labelled steroid from replicates of a plasma pool. Assay values were corrected accordingly to account for extraction losses. Interassay variability was determined by repeat measurement of a pooled internal standard and was (CV%) 7.4 (*n* = 3), 13.9 and 12.3 (*n* = 2) for E2, T and F respectively.

Plasma Vtg levels were measured by enzyme linked immunosorbent assay using the reagents and protocol as described in Watts et al. (2003). Plasma samples were diluted at 1:1,000 in assay buffer for measurements. Interassay variability was assessed by repeat measurement of a Vtg standard from the central part of the assay curve and was 13.1 (CV%, *n* = 7). Pooled internal standards were not used here due to the tendency of Vtg to denature following repeated freeze-thaw cycles. The LOD for the assay was 80 ng ml^−1^.

### RNA extraction and cDNA synthesis

Total RNA was isolated from 15 mg of hepatic tissue using the Illustra RNAspin Mini kit (GE Healthcare, Little Chalfont, UK) according to the manufacturer’s protocol. RNA yield and 260/280 purity ratio were determined using the NanoDrop 2000 (Thermo Scientific, Waltham, MA, USA). An RNA integrity number (RIN) was determined for a random sample of hepatic RNA (*n* = 24) using a 2100 bioanalyzer (Agilent, Santa Clara, CA, USA) to establish RNA quality. All RIN values were >8.5.

Four hundred nanograms of liver-derived RNA were used to synthesise cDNA for use in real-time quantitative PCR (qPCR) using the QuantiTect^®^ reverse transcription kit (Qiagen). This kit includes a DNA elimination step to remove potential contamination of PCRs by genomic DNA.

### Hepatic gene expression

Gene specific primers (Gsps) for target genes previously optimised for qPCR (Pankhurst et al., 2011) were used to amplify hepatic *vtg*, *zpc*, *zpb* (primers detect both *zpba* and *zpbb*, collectively referred to as *zpb*) and the reference gene TATA binding protein (*tbp*) transcripts (Table 1). Gsps developed by Anderson et al. (2012b) were used to amplify hepatic *erα* transcripts.

**Table 1:** qPCR gene specific primers.

Gene name	Sequence (5′–3′)		Prod. size, bp	E^a^	Source sequence
Vtg	F	AAC TTT GCC CCT GAA TTT GC	95	0.984	DQ834857
	R	GCT CTA GCC AGA CCC TCC GC			
Zpb	F	GTT TCC AGG GAT GCC ACT CT	113	0.937	AJ000664, AJ000665
	R	TGG TAG ATG GCA AAG GCA GA			
Zpc	F	GTC CCC CTG CGT ATC TTT GT	121	0.969	GU075906
	R	AAC CTG TCA CTT TGG CAT CG			
Erα	F	AAG CAT GCC GCC TCA GAA AG	150	1.003	X89959
	R	TCC TGT GCT CCA GGT CAC CA			
Tbp	F	TCC CCA ACC TGT GAC GAA CA	117	0.981	BT059217
	R	GTC TGT CCT GAG CCC CCT GA			

**Notes.**

a E, primer efficiency.

Each qPCR reaction contained 5 μl Platinum SYBR Green (Invitrogen, Waltham, MA, USA), 200 nM each primer, 1 μl cDNA template, and molecular grade water to a final volume of 10 μl. For every gene analysed no-template controls and a calibrator (which was a pool of randomly selected hepatic cDNA) were included to detect possible contamination, and control for in-between run variability, respectively. Negative reverse transcription controls were also analysed to amplify any contaminating genomic DNA.

qPCRs were conducted on a Rotor-gene 6000 series thermal cycler (Qiagen) using the cycling conditions: 50 °C–2 min; 95 °C–2 min; 40 cycles of 95 °C–15 s; 60 °C–15 s, and 72 °C–20 s (acquiring). At the end of cycle 40, a melt curve analysis was performed to confirm amplification of a single product as follows: preconditioning 72 °C–90 s, followed by a temperature gradient up to 95 °C at 1 °C per 5 s.

The suitability of using *tbp* as a reference gene for normalisation was assessed by Kruskal–Wallis analysis coupled with Bonferroni’s correction (performed on a month-by-month basis), which revealed no significant differences in *tbp* transcript abundance during the experimental period. In addition, no significant correlation was found between Cq value and sample point by Kendall’s tau correlation analysis at *p* ≤ 0.01. Thus, consistent with our previous work, liver-expressed *tbp* showed consistently high stability under the experimental conditions and was used as a reference gene for normalisation (Anderson & Elizur, 2012; Anderson et al., 2012b; Pankhurst et al., 2011). Rest©2009 (Pfaffl, Horgan & Dempfle, 2002) was used to normalise the data and calculate expression of target genes for each sample relative to the calibrator/reference sample that was analysed in each run.

### Statistical analysis

For multiple comparisons (at sample times 2, 3 and 4) of means of morphometric, plasma hormone and plasma Vtg data, one-way ANOVA with post-hoc comparison of means by Tukeys-b was performed using the SPSS (version 15.0) statistical package. Differences in gene expression levels were detected non-parametrically using the Kruskal–Wallis test coupled with Bonferroni’s Correction to reduce the risk of type 1 error. The *P* value for significance was set at 0.05 for all analyses.

## Results

### Morphometric data

**Figure 2:**
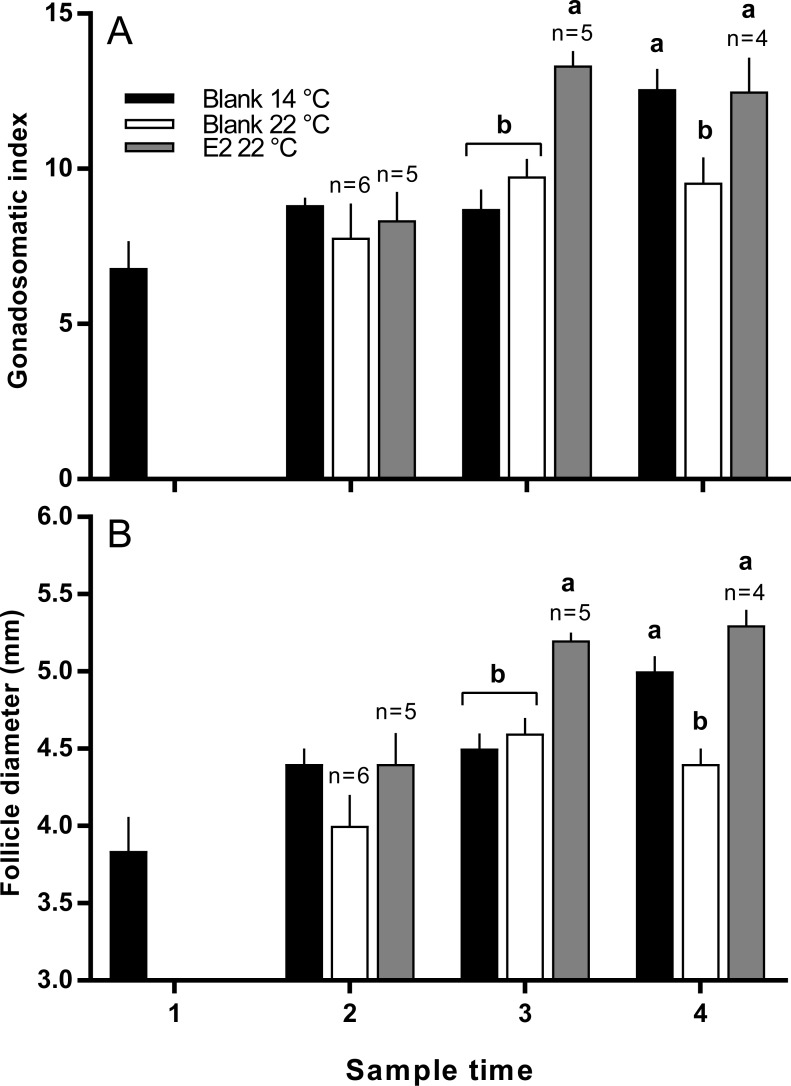
Gonadosomatic index (A) and follicle diameters (B) among maiden *S. salar* without hormone pellet implants held at 14 or 22 °C, and with E2 pellet implants held at 22 °C. Values are means + SE (*n* = 7 unless stated otherwise). Different alphabetical superscripts at each sample time denote significant differences (*P* < 0.05). Other details as for Fig. 1.

There was no significant difference in the mean length or weight of fish in any treatment group (data not shown). Similarly, there was no difference in CF among groups at any time, however there was a significant increase in gonad mass in E2-treated fish held at 22 °C in late March (data not shown). GSI was higher in E2-treated fish at sample 3 (late March) relative to other groups, and at sample 4 (early April), GSI was lowest in the untreated 22 °C (Fig. 2A). Follicle diameter followed the same pattern of significance as GSI for the duration of the experimental period (Fig. 2B).

**Figure 3:**
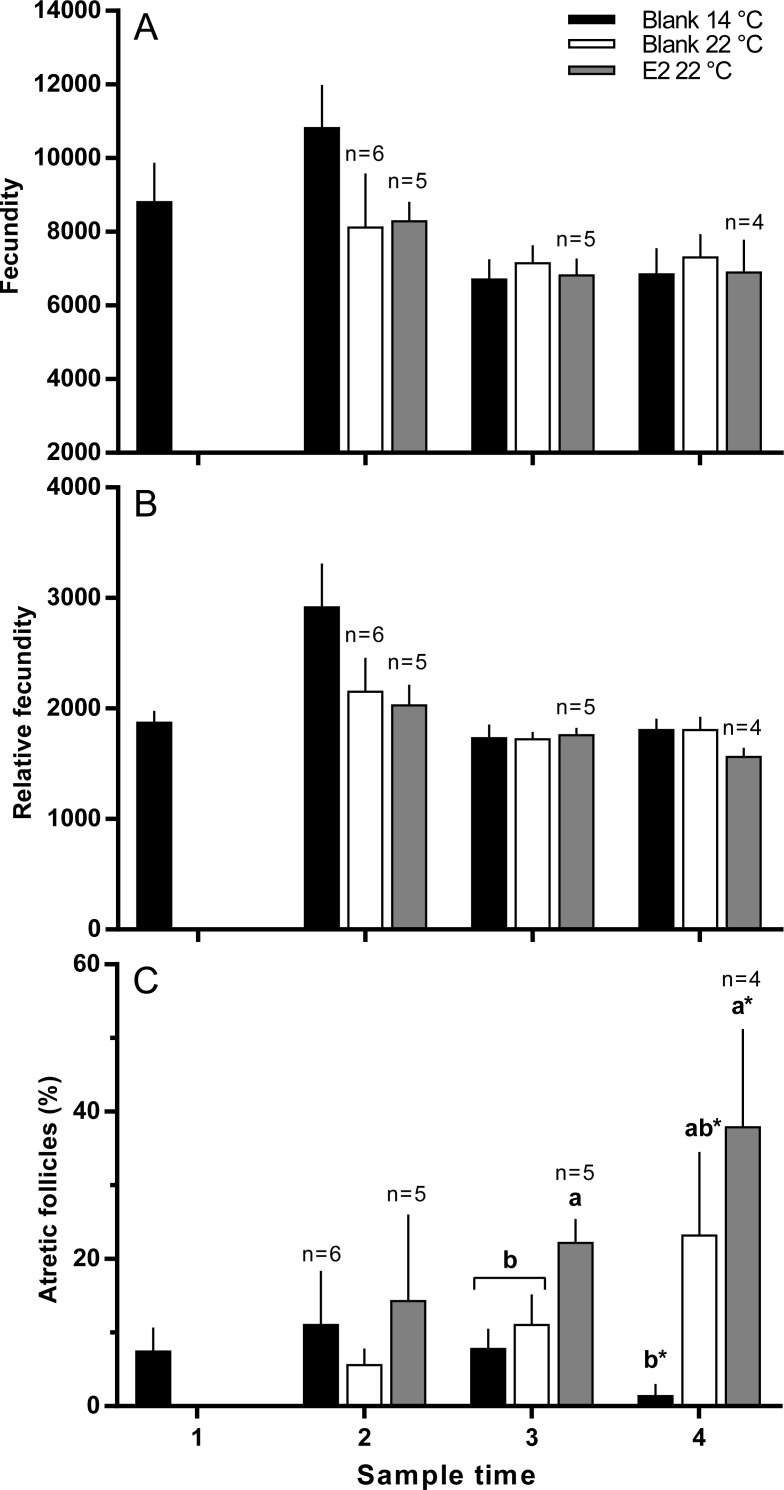
Absolute (A) and relative fecundity (B) and the proportions of atretic follicles (C) in the ovaries of maiden *S. salar* without hormone pellet implants held at 14 (open bars), or 22 °C (cross-hatched bars), and fish with E2 pellet implants held at 22 °C (black bars). Values are means + SE (*n* = 7 unless stated otherwise). Different alphabetical superscripts at each sample time denote significant differences (*P* < 0.05) except for % atresia at sample time 4 (*P < 0.057; *F* = 3.486). Other details as for Fig. 1.

There were no significant differences in absolute or relative fecundity between groups at any time (Figs. 3A and 3B). However, fish from all groups showed the presence of atretic follicles in the ovary during vitellogenesis (Fig. 3C). At sample 3, histological examination revealed higher levels of atresia in E2-treated fish held at 22 °C, than in the other two groups. At sample 4, there were high levels of atresia in both groups of fish maintained at 22 °C. However, due to high levels of variation for both groups of fish at 22 °C, only the level of atresia for E2-treated group at 22 °C was significantly different from fish maintained at 14 °C whose level of atresia appeared to decline from sample 3 to 4.

### Ovulation, egg fertility and survival

**Figure 4:**
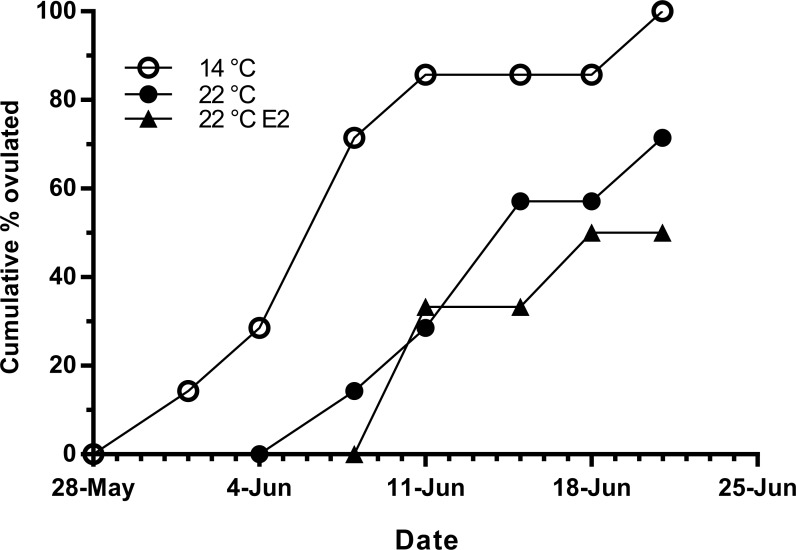
Cumulative ovulation in maiden *S. salar* spawners without hormone pellet implants held at 14 and 22 °C, and fish with E2 pellet implants held at 22 °C during autumn.

**Figure 5:**
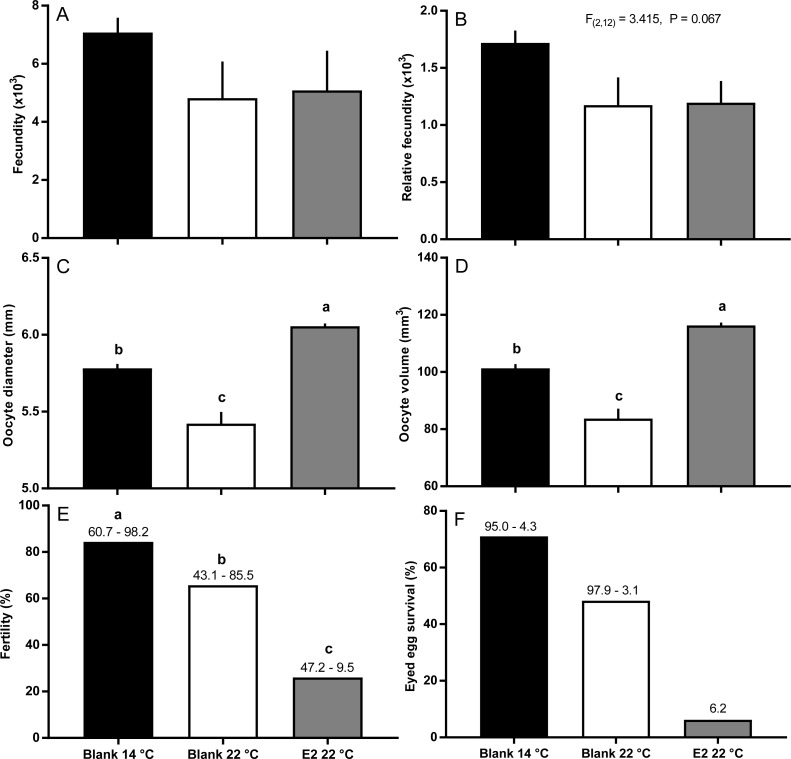
Post-ovulatory fecundity (A, B), oocyte diameter (C) and volume (D), fertility (E), and survival to eyed egg stage (F) among maiden *S. salar* without hormone pellet implants held at 14 or 22 °C, and with E2 pellet implants held at 22 °C. Values are mean + SE (or 95% confidence limits for % data) (*n* = 7, 5 and 3 for the three groups respectively; *n* = 1 for eyed egg survival in 22 °C + pellet group). Different alphabetical superscripts among sample times denote significant differences (*P* < 0.05) unless stated otherwise.

Fish held at 14 °C began ovulating in early June and had completed ovulation three weeks later (Fig. 4). The initiation of ovulation among untreated fish held at 22 °C was delayed by one week relative to 14 °C, and only 70% of those fish had ovulated by 22nd June. The pattern was similar in E2-treated fish held at 22 °C, with the additional effect that only 50% of the E2-treated fish ovulated by the end of June. For fish that did ovulate, there were no differences in absolute or relative fecundity between groups (Figs. 5A and 5B). Egg diameters and volumes were larger in E2-treated fish held at 22 °C than in both other groups, and eggs of untreated fish held at 14 °C were in turn larger than those from the control group at 22 °C (Figs. 5C and 5D). In contrast, egg fertility and survival to the eyed stage was highest in untreated fish maintained at 14 °C, reduced in untreated fish at 22 °C, and further reduced in E2-treated fish held at 22 °C (Figs. 5E and 5F).

### Plasma hormones and Vtg

Levels of plasma Fsh were low and very similar among all groups of fish (means ranged between 0.73 and 1.03 ng ml^−1^) at sample points 1, 2 and 3 (Fig S1). At sample point 4, there were no statistically significant differences in the levels of plasma Fsh, although levels were slightly higher in the 22 °C (1.61 ± 0.82 ng ml^−1^) than in the 14 °C group (0.80 ± 0.12 ng ml^−1^), and the E2-treated group at 22 °C was in-between at 1.06 ± 0.11 ng ml^−1^. Levels of plasma Lh ranged between (means) 0.55 and 0.81 ng ml^−1^ at sample points 3 and 4, and no significant differences were detected between groups (data not shown).

**Figure 6:**
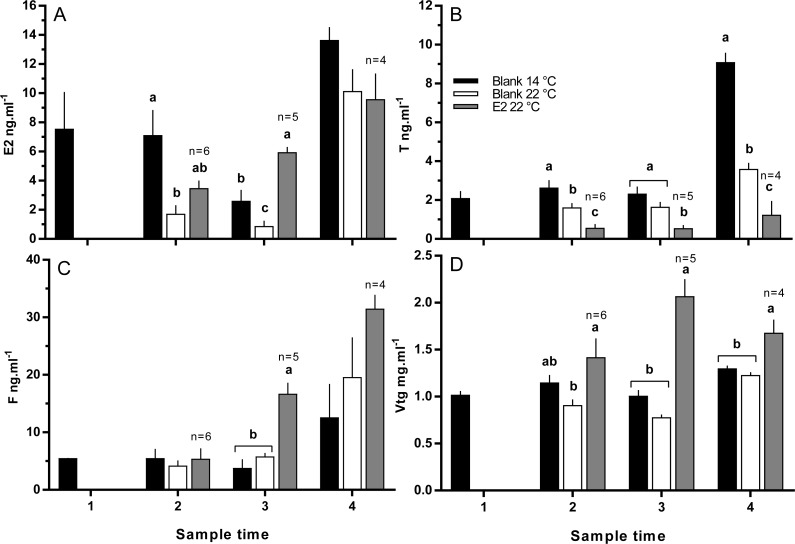
Plasma levels of estradiol (A), testosterone (B), cortisol (C) and vitellogenin (D) among maiden *S. salar* spawners without hormone pellet implants held at 14 (open bars) or 22 °C (cross-hatched bars), and fish with E2 pellet implants held at 22 °C (black bars). Values are means + SE (*n* = 7 unless stated otherwise). Different alphabetical superscripts among sample times denote significant differences (*P* < 0.05). Other details as for Fig. 1.

Plasma T levels were depressed in maiden fish reared at 22 °C relative to 14 °C at samples 2 and 4; while T levels in E2-treated fish at 22 °C were lower than all other groups at samples 2, 3 and 4 (Figs. 6A and 6B). Plasma E2 levels were suppressed in untreated fish held at 22 °C relative to 14 °C at samples 2 and 3 (Fig. 6A). E2 levels were at an intermediate value in the E2-treated group at sample 2, but were the highest of any group at sample 3. Plasma E2 levels of all groups of fish were similar at sample point 4. Plasma F levels were low (<10 ng.m1^−1^) in all fish at samples 1 and 2 (Fig. 6C). At sample 3, plasma F was elevated in E2-treated fish at 22 °C above both other groups. At sample 4, plasma F levels were similar among groups, but were generally higher than levels observed at other sample points. Plasma Vtg levels were relatively stable across sample times among fish held at 14 °C (Fig. 6D). There was no significant suppression of plasma Vtg as a result of thermal challenge at any sample time; although levels were slightly lower in fish maintained at 22 °C relative to 14 °C at samples 2 and 3. In contrast, plasma Vtg levels were elevated in E2-treated fish held at 22 °C relative to untreated fish at 22 °C at sample points 2, 3 and 4, and untreated fish at 14 °C at samples 3 and 4.

### Hepatic gene expression

**Figure 7:**
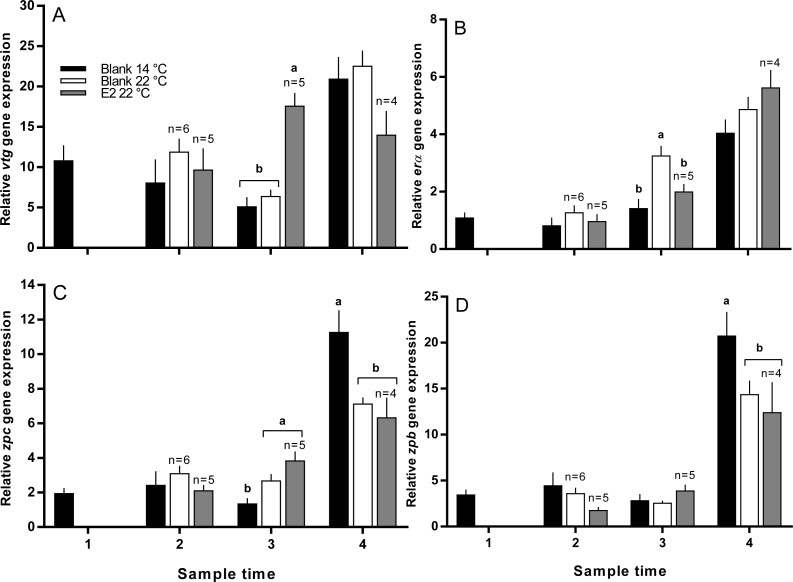
Relative gene expression of vitellogenin (A), estrogen receptor α (B), zona pellucida c and b (C and D) among maiden *S. salar* without hormone pellet implants held at 14 or 22 °C, and with E2 pellet implants held at 22 °C. Values are means + SE (*n* = 7 unless stated otherwise). Different alphabetical superscripts among sample times denote significant differences (*P* < 0.05). Other details as for Fig. 1.

Vitellogenin gene expression was similar among all groups at sample 2, and was higher in E2-treated fish at 22 °C than all other groups at sample 3 (Fig. 7A). At sample 4, there was no significant difference in *vtg* gene expression levels between groups. Zonapellucida c gene expression levels were similar among groups at sample 2, then were higher in the treated and untreated groups at 22 °C compared to the 14 °C group at sample 3 (Fig. 7C). At sample 4, thermal suppression of *zpc* gene expression was observed in the untreated and E2-treated groups at 22 °C relative to the untreated group at 14 °C. Similarly to *zpc*, *zpb* gene expression levels were not different between groups at samples 2 and 3, while thermal suppression was observed in both groups maintained at 22 °C at sample 4 (Fig. 7D). There were no significant differences in *erα* gene expression levels at any sample time, except for sample 3, where expression was higher in the 22 °C control group relative to all other groups (Fig. 7B).

## Discussion

The present study was performed to determine whether E2-therapy during vitellogenesis could offset the negative effects of thermal challenge on oocyte development, endocrine function and subsequent egg quality in maiden spawning *S. salar*. Temperature and/or hormonal treatment had no effect on fish length, weight or condition factor indicating that the experimental outcomes were not biased by these parameters. In contrast, follicle diameter and subsequently GSI were higher in E2-treated fish at 22 °C than in fish from untreated groups maintained at 14 and 22 °C at sample 3 (late March), and untreated fish maintained at 22 °C at sample 4 (April). Ovarian growth occurs primarily as a result of Vtg uptake into the oocyte (Tyler, Sumpter & Bromage, 1988), and in the present study, E2-treated fish at 22 °C displayed higher levels of plasma Vtg than the control group at 22 °C at samples 2, 3 and 4. This difference was reflected by an increase in the rate of oocyte growth in E2-treated relative to untreated fish at elevated temperature. These results demonstrate that E2-treatment helped to maintain oocyte growth at 22 °C, and imply that the mechanisms governing Vtg uptake into developing oocytes are not impaired at high temperature in salmonids. Similarly, Tyler, Sumpter & Bromage (1987) demonstrated that *in vitro* maintenance of rainbow trout (*Oncorhynchus mykiss*) follicles at 25 °C did not inhibit the uptake of Vtg from the culture medium relative to follicles held at between 0 and 20 °C (Tyler, Sumpter & Bromage, 1987).

There was no significant difference in absolute or relative fecundity between the groups during reproductive development. However, histological analysis revealed the presence of atretic follicles in all groups at all sample times. The presence of atresia in maiden fish reared at 14 °C was unexpected as our previous work has shown that atresia is either absent or affects very few follicles in maiden fish maintained at 14 °C (Pankhurst et al., 2011). Inspection of temperature records for the period prior to experimental set up showed that the maiden broodstock had experienced temperatures of between 18−22 °C during early February 2010, whereas ambient pre-set up temperatures from our previous broodstock experiments did not exceed 17 °C. This occurred due to higher-than-normal ambient temperatures during summer in combination with a delay of experimental set-up due to an approaching bushfire. Therefore, it appears that the maiden fish used in this study had some degree of unanticipated thermal exposure during February, a month that corresponds to a period of vitellogenesis that is particularly sensitive to high temperature (King, Pankhurst & Watts, 2007). It appears that fish maintained at 14 °C recovered somewhat from the pre-set up thermal exposure as evidenced by a steady decrease in the rate of ovarian atresia from sample 2 onwards. On the contrary, there was no evidence of recovery in fish reared at 22 °C, and the number of atretic follicles was generally higher in fish that received an E2 implant relative to the control group at 22 °C. The presence of atretic follicles in both groups of fish maintained at 22 °C throughout development is broadly consistent with the general effects of elevated temperature on gonadal morphology in fish (Linares-Casenave, Eenennaam & Doroshov, 2002; Pankhurst et al., 2011; Soria, Strüssmann & Miranda, 2008). Furthermore, E2-treated fish displayed an increased rate of atresia even though oocyte growth was promoted at 22 °C. In fathead minnow (*Pimephales promelas*) waterborne exposure to E2 (27.3 ng l^−1^) resulted in a high incidence of ovarian atresia (Miles-Richardson et al., 1999). However, plasma E2 levels in E2-treated fish at 22 °C did not appear to exceed 12 ng ml^−1^ which is below the concentration of plasma E2 observed in maiden fish (up to 20 ng ml^−1^) reared at 14 °C from our previous experiments that did not display significant ovarian atresia (Pankhurst et al., 2011). On the other hand, plasma F levels were significantly higher in E2-treated fish sample 3, and higher to some extent at sample 4 relative to untreated fish at 14 and 22 °C. This implies that the second hormonal implantation was perceived as stressful. In many species, stress and the subsequent production of cortisol has been linked to an increased incidence of ovarian atresia during reproductive development (Clearwater & Pankhurst, 1997; Cleary, Pankhurst & Battaglene, 2000). Therefore it is possible that atresia occurred as a part of a stress response to E2-implantation and not inappropriate levels of plasma E2 per se.

Timing of ovulation was similar between the E2-treated and control group at 22 °C, and the initiation of ovulation for these groups was delayed by 1 week relative to the control group at 14 °C. There is evidence suggesting that in salmonids, delays in ovulation at high temperature occur as a result of dopamine mediated inhibition of Lh secretion (Gillet, Breton & Mikolajczyk, 1996) and suppression of plasma maturation inducing hormone (King & Pankhurst, 2004). Furthermore, a recent study by Anderson et al. (2017) reported the thermal suppression of *star* and *3β-hsd* in the month preceding ovulation, providing more evidence to suggest the impairment of ovarian steroidogenesis. Unfortunately our sampling schedule did not capture the rise in plasma Lh that typically occurs just prior to ovulation, and basal plasma levels of Lh did not differ among groups up until at least 50 days prior to the onset on ovulation. Having said that, the timing of ovulation was similar between treated and untreated groups at 22 °C, suggesting that implantation with E2 did not significantly affect the events associated with FOM and ovulation in those fish that ovulated successfully. However, only 50 and 70% of E2-treated and untreated fish respectively ovulated at 22 °C compared to 100% in the 14 °C control group. This may indicate that thermal exposure in combination with stress related to E2-implantation inhibited ovulation in some fish, although any such inhibitory effects of temperature and cortisol were not reflected by the Lh data.

For fish that had ovulated by the end of June, absolute and relative fecundity was not affected by the experimental conditions. The largest and smallest eggs were produced by E2-treated and untreated fish respectively at 22 °C, and fish from the 14 °C group produced eggs of an intermediate size. The production of large eggs in E2-treated fish is consistent with the higher concentration of plasma Vtg and presumably Vtg uptake observed for this group at samples 3 and 4 relative to all other groups. These results indicate that treatment with E2 is able to offset the negative impacts of high temperature on egg size typically observed in *S. salar*. However, the stimulatory effect of E2-treatment on oocyte growth did not translate into an increase in egg fertility or embryo survival. In fact, egg fertility and embryo survival from E2-treated fish at 22 °C were markedly reduced relative to untreated fish maintained at 14 and 22 °C. While treatment with estrogens has been linked to reductions in egg quality at some concentrations (Lahnsteiner et al., 2006; Peters et al., 2007), it appears that in the present study, plasma E2 levels in E2-treated fish at 22 °C were generally similar to those observed in the untreated group at 14 °C that displayed a higher level of egg fertility and survival. In addition, previous research has demonstrated that plasma E2 levels are stable for between 7–14 days after implantation of a single silastic E2 pellet in other species (Pankhurst, Stacey & Peter, 1986) and juvenile *S. salar* (Anderson et al., 2012b). Therefore it is unlikely that the E2 profiles in the present study were so inappropriate that egg quality was dramatically reduced as a result E2-implantation. On the other hand, plasma F levels were elevated in the E2-treated group after receiving a second hormonal implant which is an indicator of stress as previously mentioned. Stress is able to reduce reproductive performance in many species (Schreck, 2010), and it is possible that egg quality was lower in E2-treated fish as a result of the implantation process. However, this is hard to assess in the absence of data from fish receiving a blank implant, or a hormonal treatment via less invasive means. Plasma F levels were not elevated after the first round of hormonal treatment, which suggests that use of a single implant with a slower rate of hormone release warrants investigation.

Plasma T was lower in untreated fish reared at 22 °C relative to 14 °C at samples 2 and 4, and plasma T was further suppressed in E2 treated fish relative to the 22 °C control groups at samples 2, 3 and 4. It was previously suggested that thermal inhibition of steroidogenesis may be in part caused by the downregulation of gonadal *fshr*, as this has been demonstrated in other species (Soria, Strüssmann & Miranda, 2008). However, Anderson et al. (2017) found no evidence to suggest that ovarian *fshr* was suppressed in female *S. salar* reared at 22 °C, while the expression of other factors such as *cyp11a1* were impaired, and could contribute to impairment of T production (Anderson et al., 2012a). In addition, E2 treatment is able to regulate *cyp19a1a* through a positive or negative feedback in the ovary depending on the species and stage of reproductive development (Guiguen et al., 2010). Therefore, if a positive feedback loop was functioning at high temperature, plasma T may have been further depressed in E2-treated fish due increased *cyp19a1a* mediated conversion of T to E2. However, this is unlikely as the gene expression of *cyp19a1a* is typically down-regulated in response to elevated temperature in both adult and juvenile fish (Anderson et al., 2012a; Lim et al., 2003; Van Nes & Andersen, 2006). On the other hand, exogenous treatment with natural or synthetic estrogen has been shown to down-regulate the gonadal gene expression of various steroidogenic enzymes in several species (Hogan et al., 2010; Kishida & Callard, 2001; Nakamura, Kusakabe & Young, 2009). Therefore, the additive effects of thermal and E2-treatment induced impairment of steroidogenesis may have underpinned the significant depression of T levels in E2 treated fish at 22 °C relative to all other groups.

In the present study, there was no evidence to suggest that E2-treatment affected hepatic *erα* gene expression at any point during reproductive development. This is contradictory to many studies on salmonids where E2 treatment resulted in an increase in the levels of *erα* at normal and elevated temperatures (Anderson et al., 2012b; Arukwe et al., 2002; MacKay, Raelson & Lazier, 1996; Westerlund et al., 2001). The apparent discrepancy between the present and aforementioned studies may have occurred as a result of cortisol mediated inhibition of *erα* expression. For example, in vitellogenic *O. mykiss* maintained at 12−15 °C, treatment with cortisol down-regulated the hepatic expression of *er* (Lethimonier et al., 2000). Therefore, the expected stimulatory effect of E2-treatment on hepatic *erα* may have been offset by cortisol in the present study. Nevertheless, *erα* expression in fish maintained at 22 °C was not suppressed relative to fish reared at 14 °C, consistent with previous studies (Anderson et al., 2012b; Pawlowski et al., 2000), and E2-treated fish displayed *erα* gene expression levels that were similar to those observed in untreated fish at 14 and 22 °C for the duration of the experiment (except for the 22 °C untreated group at sample 3). Therefore, even though both E2-treatment and plasma cortisol are able to modulate *erα* expression in many species, it appears that there was no net change in *erα* expression in response to either of these variables in the present study.

Treatment with E2 at 22 °C significantly increased *vtg* expression at sample 3 relative to all other groups which is broadly consistent with the role of E2 in Vtg regulation (Berg, Westerlund & Olsson, 2004; Lim, Ding & Lam, 1991). Vtg up-regulation in response to E2 demonstrates that hepatic responsiveness is maintained in adult broodstock at 22 °C, even though previous research in tilapia (*Oreochromis aureus*) and *S. salar* has shown that hepatic ER binding affinity is reduced at elevated temperature (Tan et al., 1999; Watts et al., 2005). This corroborates the findings from our previous work on E2-treated juvenile *S. salar* where vitellogenesis in fish held at 22 °C was not impaired relative to fish reared at 14 °C (Anderson et al., 2012b). At sample 2, plasma Vtg was elevated in the E2-treated group relative to the control at 22 °C prior to an increase in *vtg* at sample 3. This may indicate that treatment with E2 augmented the stability of *vtg* as observed in *O. mykiss* (Flouriot, Pakdel & Valotaire, 1996) which could make the mRNA available for translation for a longer period of time. In addition, E2 exposure in combination with high temperature could have increased the rate of *vtg* translation relative to untreated groups, as observed previously in *O. mykiss* (Mackay & Lazier, 1993). For the remaining 2 sample points, plasma Vtg was significantly higher in the E2-treated group held 22 °C than all other groups, which is consistent with an increase of *vtg* and subsequent accumulation of Vtg in blood. It is promising that E2-treatment elevated plasma Vtg levels given that fact that cortisol was also elevated in this group, and cortisol is known to have a deleterious effect on plasma Vtg levels in salmonids (Berg, Westerlund & Olsson, 2004). In addition, a recent review Guiguen et al. (2010) highlights the fact that even though E2-treatment may have a suppressive effect on steroidogenesis in some species, exogenous E2 is still able to independently induce female-specific pathways during sexual differentiation. Thus, we have also demonstrated that E2-treatment is a useful strategy for promoting female specific processes at 22 °C in adult fish, even though it is likely that gonadal steroidogenesis was thermally inhibited.

At temperatures within normal ranges, treatment with E2 strongly induces ZP synthesis in salmonids such as *O. mykiss* and Arctic charr (*Salvelinus alpinus*) (Arukwe et al., 2002; Berg, Westerlund & Olsson, 2004). However, in the present study, there was no evidence suggesting that E2-treatment at 22 °C altered the hepatic expression of either *zpb* or *zpc* at any time during oocyte development. In a previous study on juvenile salmon, we demonstrated that E2-implantation could induce *de novo* gene expression of *zpb* and *zpb* at 14 and 22 °C, however the magnitude of the response was greater at 14 °C for both genes (Anderson et al., 2012b). Therefore, there is mounting evidence to suggest that Vtg and ZPs respond differentially to E2-treatment at elevated temperature, although the cellular basis for this is currently unclear. Previous studies have shown that ER binding affinity is reduced at high temperature (Tan et al., 1999; Watts et al., 2005), and a reduction in E2 signal transduction may explain the lack of zonagenic response to E2-treatment at 22 °C. However, in our study this is unlikely to be the explanation, as ER-dependent Vtg synthesis was stimulated by E2-treatment at 22 °C, implying that this pathway is still functional to a large extent at high temperature. It has been suggested that Vtg and ZPs may be regulated by different mechanisms (Berg, Westerlund & Olsson, 2004), and the promoter regions of hepatically expressed ZP genes appear to be more complex than those found in Vtg genes (Bouter et al., 2010; Kanamori et al., 2003; Lyons et al., 1993; Teo et al., 1998). Therefore, the apparent lack of *zp* stimulation by E2 implies that factors regulating the transcription of ZP but not Vtg genes are impaired at elevated temperature in both juvenile and adult *S. salar*, although the exact mechanism by with this occurs is yet to be explored.

Loss of ZP integrity in fertilised eggs has been linked to reduced egg viability in farmed Atlantic and chinook salmon (*Oncorhynchus tshawytscha*) (Barnes et al., 2003; King et al., 2003). In *S. salar*, the thermal sensitivity of hepatic *zp* expression during oocyte development has been previously demonstrated, and it is thought that ZP damage is present prior to spawning as a consequence (King et al., 2003; Pankhurst et al., 2011). Therefore, the reduction of *zp* expression in both groups of fish maintained at 22 °C relative to 14 °C at sample 4 is consistent with our previous work, and could have contributed to the lower egg fertility and embryo survival observed in both groups maintained at 22 °C. However, at this time it is not clear whether only ZP transcription is depressed, or whether down-stream *zp* translation and/or protein assembly around the oocyte are also influenced by temperature.

## Conclusions

The present study trialled E2-therapy as a novel means of elevating plasma E2, maintaining downstream E2-dependent endocrine function and promoting high egg quality in maiden *S. salar* reared at elevated temperature. Treatment with E2 at 22 °C stimulated *vtg* expression and subsequent protein synthesis which indeed promoted oocyte growth relative to untreated fish at 14 and 22 °C, but did not improve egg fertility and embryo survival. This could be due to the fact that treatment with E2 did not offset the negative effects of maintenance at high temperature on the hepatic expression of ZP genes during oocyte development. There was no evidence to suggest that thermal impairment of ZP genes occurs as a result of lowered hepatic *erα* expression and thus, the cellular basis for the differential response of Vtg and ZP genes to E2-treatment is currently unclear. In addition, the apparent stress caused by the double implant protocol in the present study seems to have resulted in a further reduction in egg quality in E2-treated fish maintained at 22 °C, and therefore may not be an appropriate means of hormonal delivery.

##  Supplemental Information

10.7717/peerj.3897/supp-1 Figure S1 Plasma FSH data Mean ± SE plasma Fsh levels in maiden spawning female Atlantic salmon throughout reproductive development. Values that were below the limit of reliable detection (LOD) were graphed as 0.6 ng/ml (equal to LOD). Black, grey, and white bars represent maiden fish reared at 14 °C (no hormonal treatment), 22 °C (no hormonal treatment), and E2-treated fish maintained at 22 °C respectively.

10.7717/peerj.3897/supp-2 Data S1 All raw data

## Additional Information and Declarations

### Data Availability

The following information was supplied regarding data availability: The raw data has been provided as a Supplemental File.
