# Supplementary material for: Estrogen therapy offsets thermal impairment of vitellogenesis, but not zonagenesis, in maiden spawning female Atlantic salmon (Salmo salar)
Source: PeerJ. 2017 Nov 2;5:e3897. doi: 10.7717/peerj.3897 (PMC5671662; doi:10.7717/peerj.3897)
Supplement: Figure S1 — Mean ± SE plasma Fsh levels in maiden spawning female Atlantic salmon throughout reproductive development. Values that were below the limit of reliable detection (LOD) were graphed as 0.6 ng/ml (equal to LOD). Black, grey, and white bars represent maiden fish reared at 14 °C (no hormonal treatment), 22 °C (no hormonal treatment), and E2-treated fish maintained at 22 °C respectively. [file peerj-05-3897-s001.docx]

Levels of plasma Fsh were low and very similar among all groups of fish (means ranged between 0.73 and 1.03 ng/ml, Figure S1).


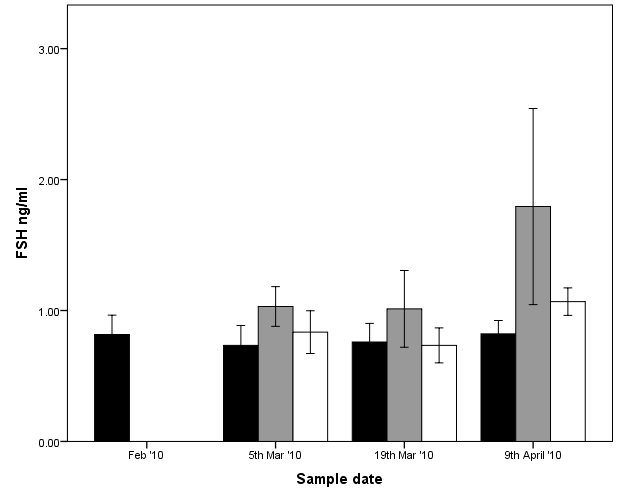


**Figure S1**. Mean ± SE plasma Fsh levels in maiden spawning female Atlantic salmon throughout reproductive development. Values that were below the limit of reliable detection (LOD) were graphed as 0.6 ng/ml (equal to LOD). Black, grey, and white bars represent maiden fish reared at 14 °C (no hormonal treatment), 22 °C (no hormonal treatment), and E2-treated fish maintained at 22 °C respectively.
